# Efficient One-Step Knockout by Electroporation of Ribonucleoproteins Into Zona-Intact Bovine Embryos

**DOI:** 10.3389/fgene.2020.570069

**Published:** 2020-09-07

**Authors:** Luiz Sergio Almeida Camargo, Joseph R. Owen, Alison L. Van Eenennaam, Pablo Juan Ross

**Affiliations:** ^1^Brazilian Agricultural Research Corporation (Embrapa Dairy Cattle), Juiz de Fora, Brazil; ^2^Department of Animal Science, University of California, Davis, Davis, CA, United States

**Keywords:** embryo, genome editing, CRISPR, Cas9, OCT4 gene

## Abstract

Somatic cell nuclear transfer or cytoplasm microinjection have been used to generate genome-edited farm animals; however, these methods have several drawbacks that reduce their efficiency. This study aimed to develop electroporation conditions that allow delivery of CRISPR/Cas9 system to bovine zygotes for efficient gene knock-out. We optimized electroporation conditions to deliver Cas9:sgRNA ribonucleoproteins to bovine zygotes without compromising embryo development. Higher electroporation pulse voltage resulted in increased membrane permeability; however, voltages above 15 V/mm decreased embryo developmental potential. The zona pellucida of bovine embryos was not a barrier to efficient RNP electroporation. Using parameters optimized for maximal membrane permeability while maintaining developmental competence we achieved high rates of gene editing when targeting bovine OCT4, which resulted in absence of OCT4 protein in 100% of the evaluated embryos and the expected arrest of embryonic development at the morula stage. In conclusion, Cas9:sgRNA ribonucleoproteins can be delivered efficiently by electroporation to zona-intact bovine zygotes, resulting in efficient gene knockouts.

## Introduction

The rapid advance of CRISPR/Cas9 technology has enabled the efficient generation of gene edited animals by one-step embryo manipulation (Wang et al., 2013). The CRISPR/Cas9 system, consists of a complex formed by Cas9 endonuclease, which cuts the target DNA site creating a double-strand break (DSB) and single guide RNA (sgRNA) which interacts with Cas9 and provides target recognition by simple Watson-Crick sequence complementarity (Jinek et al., 2012). In the presence of the NGG protospacer motif upstream of the sgRNA recognition sequence, SpCas9 introduces a DSB at the specific genomic location. DSBs are typically repaired by cells or embryos using one of two repair mechanisms: non-homologous end-joining (NHEJ) or homologous-directed repair (HDR). NHEJ can sometimes be error prone, often introducing insertion or deletion (indel) mutations in the repaired region, which if resulting in a frame-shift mutation at a protein coding region can effectively generate a loss-of-function mutation or gene knock-out (KO). HDR uses a homologous region of DNA to repair the DSB with high fidelity, which offers the opportunity of providing the cells with an artificial nucleic acid repair template for introducing a specific mutation, which can range from a single SNP up to introduction of a whole gene (Cong et al., 2013).

Gene editing technologies can find applications ranging from basic research to gene therapy (Doudna and Charpentier, 2014; Knott and Doudna, 2018). In livestock, gene editing could be used to generate genetically engineered animals to synthetize recombinant pharmaceutical drugs (Oishi et al., 2018), or organ donors for xenotransplantation (Niemann and Petersen, 2016; Cowan et al., 2019). Moreover, genome editing can be utilized to increase disease resistance (Burkard et al., 2017), or the frequency of alleles or polymorphisms associated to favorable traits (Jenko et al., 2015; Hickey et al., 2016; Tait-Burkard et al., 2018) such as heat tolerance, milk and/or meat production/composition.

In order to generate genome-edited animals, gene editing systems has been used to edit the genome of somatic donor cells which have then been used to produce live animals through somatic cell nuclear transfer (SCNT). However, this approach has limitations due to the low efficiency of SCNT for generating healthy cloned animals (Akagi et al., 2014; Vajta, 2018). The CRISPR/Cas9 system has also been delivered to *in vitro*-fertilized zygotes by cytoplasmic or pronuclear microinjection, avoiding the issues associated with SCNT. While the efficiency of producing live animals using this approach is higher than SCNT, embryo manipulation requires special skills and expensive equipment, as well as being laborious and time-consuming. Moreover, CRISPR/Cas9 microinjection of zygotes frequently results in genetic mosaicism, which has been reported in several species (Mianné et al., 2017; Lamas-Toranzo et al., 2018), including rabbits (Wan et al., 2019), mice (Yen et al., 2014; Horii and Hatada, 2017), pigs (Sato et al., 2015), and cattle (Bevacqua et al., 2016).

An alternative to cytoplasmic microinjection is zygote electroporation. Electroporation has been shown to deliver genome editing reagents, including Cas9:sgRNA ribonucleoproteins (RNP), to mouse, rat and pig zygotes with reasonable efficiency (Kaneko, 2017; Teixeira et al., 2018; Hirata et al., 2019). Recent reports demonstrated that electroporation could be used to deliver RNP into bovine zygotes; however, this came at the cost of compromised embryo development resulting in a decreased blastocyst rate (Miao et al., 2019; Namula et al., 2019). In this study, we aimed to optimize electroporation conditions to deliver Cas9:sgRNA RNPs to bovine zygotes to introduce gene silencing mutations and to evaluate the resulting embryonic phenotype.

## Materials and Methods

### Experimental Design

This study was composed of five complementary optimization experiments. The first experiment evaluated the effect of increasing voltages (0, 10, 15, 20, 25, and 30 V) on permeability of bovine zygotes to 3 kDa tetramethylrhodamine-labeled dextran (Thermo Fischer Scientific, Walthan, United States). The second experiment evaluated the effect of voltages (0, 15, and 20 V) on embryo development. Zygotes were electroporated in OptiMEM (Thermo Fischer Scientific) and cleavage and blastocyst rates were evaluated. The third experiment assessed the effect of electroporation (15 V) with two different RNPs concentrations (2.15 μM = 100:50 ng/μL and 4.3 μM = 200:100 ng/μL Cas9:sgRNA; 1:2.5 molar ratio) on embryo development and mutation rate. For this experiment, sgRNA targeting the zinc finger protein X-linked (*ZFX*) gene were used. The fourth experiment evaluated the effect of zona drilling (laser ablation of small points of the zona pellucida) before electroporation with RNPs (200:100 ng/μL Cas9:sgRNA *ZFX*) on embryo development and mutation rate. The fifth experiment evaluated the efficiency of the optimized RNP electroporation protocol by targeting an embryo specific gene (octamer-binding transcription factor 4; *OCT4*, a.k.a. POU class 5 homeobox) that allows for phenotypic assessment of the induced mutations. This experiment included three groups: control, representing embryos not subjected to electroporation; Electroporated controls, embryos electroporated with RNPs targeting a gene not required for development (stearoyl-CoA desaturase; *SCD1*); and OCT4-KO, embryos electroporated with RNPs targeting exon 2 of *OCT4* (a gene required for expanded blastocyst formation). In both electroporation groups, the RNP concentration was 200:100 ng/μL Cas9:sgRNA. Cleavage and blastocyst rates were recorded for each group. Embryo genotyping was performed in day 6 morulas. Embryos (32 or more cells) at day 6 and day 8, 144, and 192 post fertilization (hpf), respectively, were fixed and immunostained to evaluate the presence of OCT4 protein. Experiments 1–4 were carried out with parthenogenetic embryos, whereas experiment 5 was carried out with *in vitro*-fertilized embryos.

### Single Guide RNAs (sgRNAs)

Single guide RNAs were designed to target *ZFX* (5′- TCTTACAAGGGTGATAGTAC), *SCD1* (5′- CTGACTTACC CGCAGCTCCC) and *OCT4* (5′- GATCACACTAGGATATAC CC) genes. These sgRNA were produced by *in vitro* transcription (*ZFX*) using the AmpliScribe T7-Flash Transcription kit (Lucigen, Palo Alto, CA) and purified using the MEGAclear Transcription Clean-Up kit (Thermo Fischer Scientific, Chicago, IL), or by Synthego Corporation, Redwood City, United States (*SCD1* and *OCT4*).

### Oocytes Recovery, *in vitro* Maturation (IVM), Parthenogenesis and *in vitro* Fertilization (IVF)

Ovaries were obtained from a commercial cattle slaughterhouse (Cargill, Fresno, United States) and transported to the laboratory in saline solution at 34–36°C. Follicles with 3–8 mm diameter were aspirated and cumulus-cell oocytes (COC) complexes with homogeneous cytoplasm and compact layers of cumulus cells were selected. IVM was performed for 21–22 h in BO-IVM medium (IVF Bioscience, Fallmouth, United Kingdom) at 38.5°C, 5% CO_2_ and humidified air. Parthenogenetic activation for experiments 1–4 was induced in denuded oocytes by 5 μM ionomycin (Sigma Aldrich, Saint Louis, United States) incubation during 4 min at 38.5°C in air followed by 2 mM 6-(Dimethylamino) purine (6-DMAP; Sigma) for 4 h at 38.5°C, 5% CO_2_ in atmospheric air. *In vitro* fertilization was performed by incubating COCs with 1 × 10^6^ spermatozoa/mL in BO-IVF medium (IVF Bioscience) for 17–18 h at 38.5°C, 5% CO_2_ in humidified air.

### Laser Zona Drilling

Presumptive zygotes were denuded of cumulus cells by vortexing for 3 min and placed in a warmed 20 μL drop of SOF Hepes medium under mineral oil and “zona drilling” was performed using an inverted microscope equipped with laser system (Saturn Laser System, Research Instruments Ltd., Cornwall, United Kingdom). The zona pellucida was ablated at two points using pulses of laser beam set to 0.5–0.6 ms in order to make holes with ∼16 μm diameter. Afterward, zygotes were washed twice in SOF Hepes medium before undergoing electroporation.

### Electroporation and Embryo Culture

**FIGURE 1:**
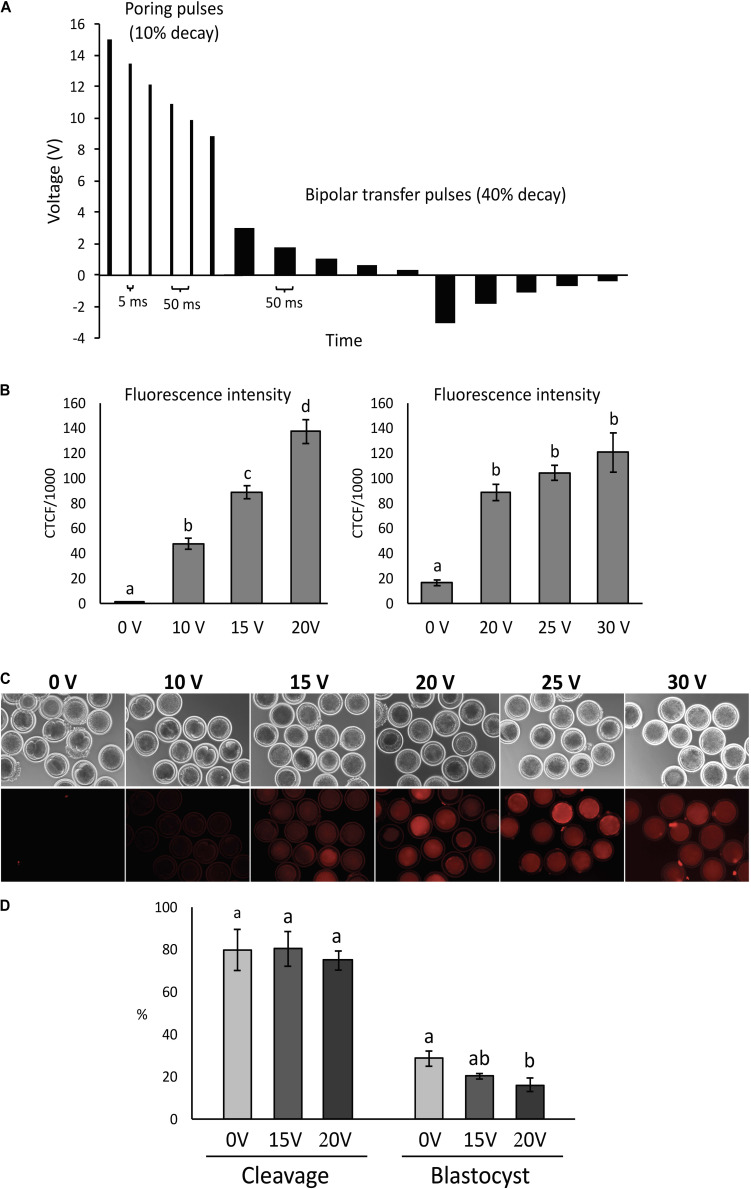
Condition optimization for efficient electroporation of bovine zygotes. **(A)** Diagram depicting the electroporation settings used in the study. **(B)** Fluorescence intensity of parthenogenetic bovine zygotes after electroporation in the presence of tetramethylrhodamine-labeled dextran (*n* = 54 zygotes). ^*a*– *d*^Different letters indicate statistically significant differences (*P* < 0.001). CTCF, corrected total cell fluorescence. **(C)** Representative images of zygotes after electroporation at different voltages. **(D)** Development of parthenogenetic zygotes after sham electroporation at different initial voltages. ^*a,b*^Different letters indicate statistically significant Intensity of differences (*P* < 0.05). Experiment replicated four times. Sample size per group: 0 V = 114 zygotes; 15 V = 89 zygotes; and 20 V = 84 zygotes.

Electroporation of denuded presumptive zygotes was performed using the Nepa21 electroporator system (Nepagene, Chiba, Japan) and a glass slide with 1 mm gap between electrodes (BEX, Japan). Embryos were electroporated following activation or *in vitro* fertilization. Poring pulses were set to different initial voltage (0, 10, 15, 20, 25, or 30 V/mm, accordingly to the experiment), always including 6 pulses of 1.5 ms at 50 ms intervals and a 10% decay rate of successive pulses. Transfer pulses were set at 3 V/mm, 5 pulses of 50 ms at 50 ms interval with 40% decay rate and positive/negative polarity (Figure 1A). RNPs solution with 200:100 ng/μL Cas9:sgRNA was prepared with 4.8 μL Cas9 protein (PNA Bio, Thousand Oaks, United States) stock solution (500 ng/μL) and 6 μl sgRNA stock solution (200 ng/μL) plus 1.8 μL OptiMEM to have a final work solution with 12 μL containing 200 ng/μL Cas9 protein + 100 ng/μL sgRNA. That solution was diluted with plus 12 μL OptiMEM to make the 100:50 ng/μL RNP solution. RNP solution was mixed and kept on ice for 5–10 min before using for electroporation. Electrode gap was filled with 3–4 μL and checked the impedance. Oocytes were washed three times in OptiMEM and once in RNP solution before electroporation. Pools of 30–40 zygotes were placed in line between the electrodes using a mouth-pipette and electroporated at room temperature. Afterward, zygotes were collected and washed three times in SOF Hepes followed by two times in BO-IVC medium (IVF Bioscience) and then cultured in BO-IVC medium at 38.5°C, 5% CO_2_, 5% O_2_, and 90% N_2_ in humidified air. Supplementation with fetal bovine serum (2.5%) was performed at 72 h post activation/IVF when cleavage rate was recorded. Blastocyst rate was recorded at 168–192 h post activation/IVF.

### Analysis of Zygotes Permeability to Dextran

For experiment 1, parthenogenetic zygotes were electroporated with 2 mg/mL of tetramethylrhodamine-labeled dextran diluted in DPBS and presence of the dye in the cytoplasm was evaluated by epi-fluorescence microcopy 20–30 min after electroporation. Corrected total cell fluorescence (CTCF) was calculated and means compared among treatment groups.

### Embryo Lysis and Sequencing

Single embryos were collected at morula (Experiment 5) or blastocyst (Experiments 3–4) stage and lysed in 10 μL lysis buffer (Lucigen, Palo Alto, CA, United States) at 65°C for 6 min and 98°C for 2 min. PCR reactions were performed in two rounds with 35 cycles each. First PCR was composed of 9.2 μL embryo lysis and 10 μL Master Mix (GoTaq Hot Start Green Master Mix, Promega, Madison, United States) at 0.8 μL of 10 μM primers (Table 1) in DNAse/RNAse free water. Second round of PCR was composed of 5 μL from first PCR, 4.2 μL of water, 10 μL Master Mix and 0.8 μL of 10 μM primers in DNAse/RNAse free water. PCR conditions included one cycle at 95°C for 3 min followed by 35 cycles of 95°C for 30 s, primer annealing temperature for 30 s (*ZFX*: 60°C; *OCT4*: 54°C) and elongation at 72°C for 30 s, and then 1 cycle at 72°C for 5 min. PCR products were run in a 1% agarose gel and bands were extracted and purified (Qiaquick Gel extraction kit, Qiagen, Hilden, Germany) for Sanger sequencing. Sequencing was performed by services provided by Genewiz (South Plainfield, NJ, United States). Mutations were analyzed by ICE CRISPR Analysis Tool (Synthego) and multiple sequence alignment (SNAPGene, GSL Biotech LLC, Chicago, United States). Indel rate was calculated based on the proportion of embryos with insertions/deletions vs. embryos sequenced.

**TABLE 1:** PCR primer sequences spanning the *OCT4* and *ZFX* sgRNA target sites.

**Gene symbol**	**Primer sequence (5′–3′)**	**Fragment size (bp)**	**Gene ID**
*OCT4*	F-AGAGGGGGTGAGGTGGATAG	854	282316
	R-CCAGTATCAGGGGGACAATG		
*ZFX*	F-AGCAGTGCTTCCAAACTTGAG	520	280961
	R-GATGAGAGCTTATGTAACTGTTGG		

### Embryo Immunostaining

Embryos with 32 or more cells at 144 h post IVF were fixed in 4% paraformaldehyde and permeabilized with 1% Triton X-100 in PBS. Samples were blocked with 1% BSA and 10% normal donkey serum in DPBS and incubated overnight with goat anti-OCT4 primary antibody (1:300; OCT3/4 antibody, Santa Cruz Biotechnology, Santa Cruz, United States). After extensive washing, embryos were incubated for 1 h with anti-goat IgG Alexa 568 secondary antibody (1:500; Invitrogen, United States) and 20 min with 10 μg/mL Hoechst 33342. Samples were observed using an epi-fluorescence microscope (Revolve, Echo, San Diego, United States). Number of cells per embryo showing expression Hoechst and/or Alexa 568 fluorescence was recorded and means compared between treatments.

### Statistical Analysis

Each experiment was independently repeated at least three times. The number of embryos analyzed for each experiment is provided in Supplementary Table 1. Developmental data, CTCF, number of total cells and cells expressing OCT4, were analyzed by analyses of variance and means compared by Tukey’s test. Results are shown as mean ± S.E.M. Proportion of embryos with indels were analyzed by Chi-square. Differences were considered significant at the 95% confidence level (*P* < 0.05).

## Results

### Experiment 1

Zygotes were electroporated with tetramethylrhodamine-labeled dextran and fluorescence intensity was measured to assess the effect of electroporation voltage on membrane permeability. Comparisons were performed at 0, 10, 15, and 20 V, followed by 0, 20, 25, and 30 V.

Fluorescence increased (*P* < 0.001) with increasing voltage up to 20 V, but there was no difference (*P* > 0.05) from 20 to 30 V (Figures 1B,C).

### Experiment 2

Parthenogenetic zygotes were electroporated with 0, 15, and 20 V in OptiMEM medium only and cleavage and blastocyst rate were compared. There was no significant effect of voltage (*P* > 0.05) on cleavage rates. Blastocyst rates were similar between embryos electroporated at 15 V compared to 0 V controls (28.5 ± 3.6% and 20.2 ± 1.3%, respectively), but were significantly reduced in embryos exposed to 20 V (16.2 ± 3.2%) compared to controls (*P* < 0.05; Figure 1D).

### Experiment 3

**FIGURE 2:**
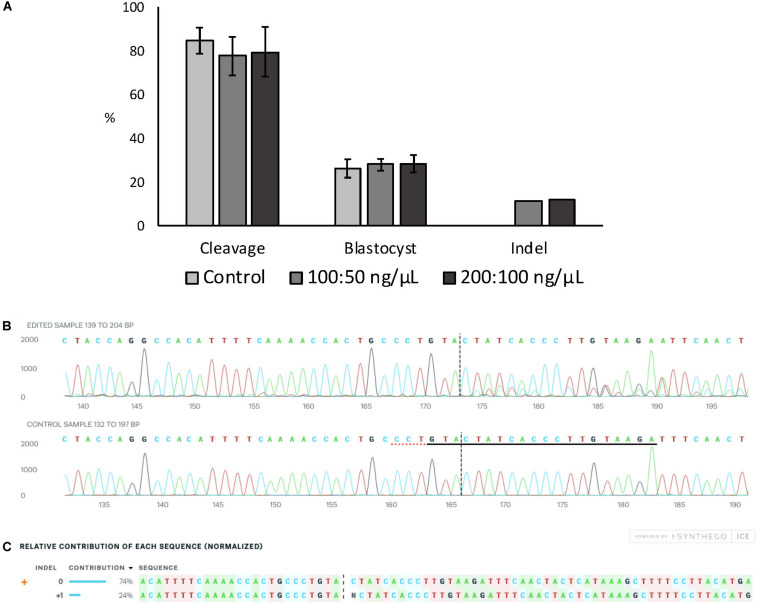
Effect of Cas9:sgRNA concentration on electroporation efficiency of bovine zygotes. **(A)** Cleavage and blastocyst rates (based on number of presumptive zygotes cultured) and indel rate (based on number of blastocysts sequenced) after electroporation of bovine zygotes with RNPs targeting the ZFX gene. No difference between groups was observed (*P* > 0.05). The experiment was replicated three times. Sample size per group: control (no electroporation) = 112 zygotes; 100:50 ng/μL = 103 zygotes, and 200:100 ng/μL = 101 zygotes. Blastocysts sequenced: 100:50 ng/μL = 27; 200:100 ng/μL = 25. **(B)** Trace file provided by ICE software of a representative blastocysts electroporated with 15 V and 200:100 ng/μL Cas9:sgRNA. The sgRNA *ZFX* sequence is underlined in black and the PAM sequence is denoted by a dotted red underline in the control sample. **(C)** Relative contribution of each sequence identified by ICE in the same representative embryo. The insertion of one nucleotide was derived from one sequence with a contribution of 24%. Expected Cas9 cut site is shown by black vertical dotted lines in **(B,C)** figures.

Embryo development and indel rate were evaluated when electroporation at 15 V was performed using two different Cas9:sgRNA RNPs concentrations (100:50 and 200:100 ng/μL of Cas9:sgRNA). The *ZFX* gene was targeted using a previously validated sgRNA (data not shown). There was no effect on cleavage or blastocyst rates between the Cas9:sgRNA concentrations evaluated, nor there was any differences in the achieved indel rate (Figure 2A). Figures 2B,C show ICE analysis of sequencing data of a representative embryo displaying the insertion of one nucleotide.

### Experiment 4

**FIGURE 3:**
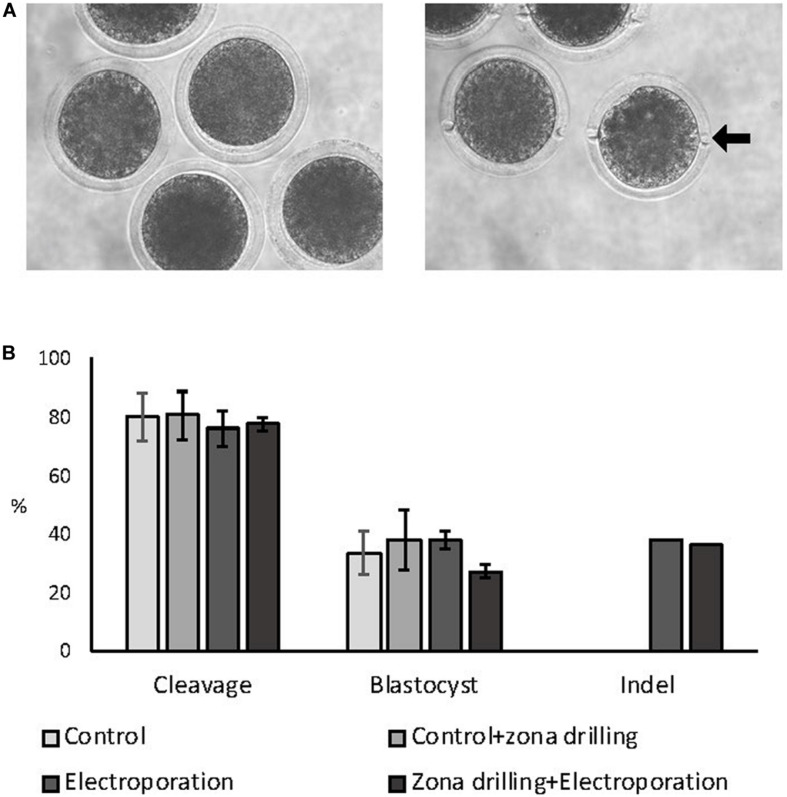
Effect of zona drilling on electroporation efficiency of bovine zygotes. **(A)** Picture of intact (left) and zona drilled (right) embryos. Arrow indicates one of the two holes made in the zona pellucida of each embryo. **(B)** Cleavage and blastocyst rates (based on number of presumptive zygotes cultured) and indel rate (based on number of blastocysts sequenced) after electroporation with RNPs targeting the *ZFX* gene. No difference between groups were observed (*P* > 0.05). The experiment was replicated three times. Sample size: control (no electroporation and no zona drilling) = 69 zygotes; control+zona drilling = 69 zygotes; intact electroporation = 45 zygotes; zona drilling+electroporation = 98 zygotes. Blastocysts sequenced: electroporation = 16; zona drilling+electroporation = 25.

Zygotes were electroporated with 15 V using 200:100 ng/μL of Cas9:sgRNA RNPs targeting the *ZFX* gene in intact and zona-drilled zygotes (Figure 3A). No differences in blastocyst rate or CRISPR-induced indel rates were observed between electroporated embryo groups (*P* > 0.05; Figure 3B), with electroporated embryo groups presenting similar developmental rates to controls (*P* > 0.05; Figure 3).

### Experiment 5

Finally, we evaluated the efficiency of CRISPR/Cas9 RNPs electroporation (15 V with 200:100 ng/μL of Cas9:sgRNA) for inducing a loss-of-function mutation to a gene required for blastocyst formation (*OCT4*), thus allowing phenotypic assessment during *in vitro* culture. For this purpose, we used a sgRNA that was previously reported to efficiently knockout bovine *OCT4* after zygote cytoplasm microinjection (Daigneault et al., 2018). A non-electroporated and an electroporated control group, with RNPs targeting a gene not related to early embryo development (*SCD1*), was included in each experiment.

**FIGURE 4:**
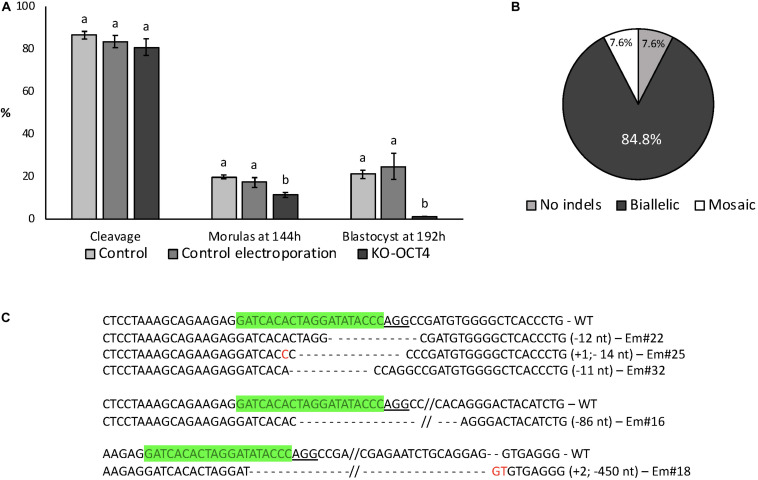
Developmental capacity of zygotes electroporated with RNPs targeting *OCT4*. **(A)** Embryo development until blastocyst stage. Control: no electroporation; Control electroporation: electroporation with RNPs targeting *SCD1*; KO-OCT4: electroporation with RNPs targeting *OCT4*. ^*a,b*^Different letters within developmental stage indicate statistically significant differences (*P* < 0.05). Sample size for cleavage and morulas at 144 h (five replicates): control = 167; control electroporation = 117; and KO-OCT4 = 220. Sample size for blastocyst (three replicates): control = 81; control electroporation = 55; and KO-OCT4 = 87. **(B)** Genotyping of morulas electroporated with RNPs to knockout *OCT4* (*n* = 13). **(C)** Alignment of sequences from representative morula stage embryos targeted for OCT KO. WT, wildtype; Em, Embryo; green sequences, sgRNA; underlined sequence, PAM; red nucleotide, insertion.

Electroporation with RNPs targeting *OCT4* (KO-OCT4 group) did not affect cleavage rate (*P* > 0.05) but significantly decreased the proportion of morulas at 144 hpf (*P* < 0.05) and blastocysts at 192 hpf (*P* < 0.01; Figure 4A). Indeed, only one blastocyst was found in the KO-OCT4 group at 192 hpf from a total of 87 embryos evaluated. There was no effect of control electroporation on cleavage or blastocyst formation (*P* > 0.05; Figure 4A).

Of 13 KO-OCT4 morulas evaluated, 12 (92.3%) presented indel mutations, with most of the mutated embryos (11/12) having biallelic mutations (Figure 4B). The other mutated morula was considered mosaic based on chromatogram analysis of PCR products. Sequence alignment showed that deletions were more frequent than insertions and ranged from 2 to 450 nucleotides (Figure 4C).

**FIGURE 5:**
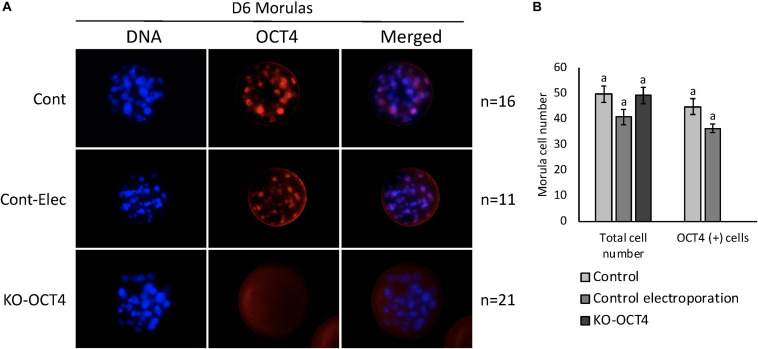
Zygote electroporation with RNPs targeting *OCT4* eliminated OCT4 expression in morula stage embryos. **(A)** Immunofluorescence analysis of OCT4 expression in morulas collected 144 h post *in vitro* fertilization (D6) in control (Cont), control electroporation (Cont-Elect) and OCT4-targeting RNPs electroporation (KO-OCT4) groups. **(B)** Number of total cells and cells expressing OCT4 (OCT4^+^) in morulas collected 144 h after *in vitro* fertilization. n: number of embryos evaluated in each group. No statistical difference in total cell number detected between groups (*P* > 0.05). No statistical difference in OCT4^+^ cell between control groups. No OCT4^+^ cells found in any morula analyzed in KO-OCT4 group.

No significant difference between total cell number in morulas collected at 144 hpf was observed between control, control electroporation and KO-OCT4 groups (*P* > 0.05; Figures 5A,B). OCT4 immunostaining was negative in all morulas evaluated from the KO-OCT4 group (Figures 5A,B), whereas controls were OCT4 positive with a similar number of OCT4 positive cells (*P* > 0.05) between control groups (Figures 5A,B). The single blastocyst found in the KO-OCT4 group was at an early stage, with only 76 cells and expression of OCT4 was absent (Supplementary Figure 1), in contrast to control and control electroporated embryos that averaged 101 ± 8.6 and 102 ± 8.7 cells, respectively, and all expressed OCT4 (Supplementary Figure 1).

## Discussion

We report an optimized electroporation condition that allowed highly efficient gene KO, as demonstrated by embryo genotyping, lack of gene product, and expected developmental phenotype (embryonic arrest). To limit the detrimental effect of electroporation on embryo development, voltage had to be kept at 15 V/mm, which was sufficient to achieve high membrane permeabilization and efficient delivery of CRISPR/Cas9 RNPs.

Using a 3 kDa tetramethylrhodamine-labeled dextran, we determined effective conditions for membrane permeabilization, as had previously been done in rat embryos (Kobayashi et al., 2018). We found that voltage as low as 10 V allows delivery of dextran, with membrane permeation to the dye increasing up until 20 V, without further improvement with higher voltage levels. While 20 V pulses maximized membrane permeabilization, this voltage level impaired bovine embryo development to the blastocyst stage. Similar results were previously reported, where pulses of 20, 25, and 30 V resulted in lower bovine blastocyst development (Miao et al., 2019). Under our conditions, 15 V, which achieved significant membrane permeabilization, did not affect embryo development and was chosen as optimal voltage for electroporation. A recent study also observed that 15 V was the highest voltage at which bovine embryos could be electroporated without affecting development to blastocyst stage (Namula et al., 2019).

Electroporation of rat and mouse zygotes has been shown to be effective with 40–50 V (Kaneko, 2017; Kobayashi et al., 2018; Teixeira et al., 2018), which is higher than the 15 V used in bovine zygotes. It has been shown that the size of a cell is an important parameter influencing electroporation (Agarwal et al., 2007). Reversible membrane permeabilization on larger cells can be achieved at lower voltages than what is required for smaller cells (Kandušer et al., 2006). Bovine oocytes and zygotes are larger (∼120 μm diameter) (Fair et al., 1997) than those of rats and mice (∼70 μm) (de Wolff-Exalto and Groen-Klevant, 1980; Eppig, 1996), suggesting that lower voltages could be effective for bovine embryos, as found in our study.

Our electroporation conditions use a series of high-voltage (HV) pulses followed by a series of low-voltage (LV) pulses with polarity inversion (poring and transfer pulses, respectively). Combination of HV with LV has been shown to increase the transfection of eukaryotic cells with plasmid DNA or siRNA (Stroh et al., 2010), especially when using low DNA concentration (Kandušer et al., 2009; Čepurnienë et al., 2010). While HV pulses are important to create pores for permeabilization, the LV pulses allow the DNA to be electrophoretically dragged into the cell (Sukharev et al., 1992). In addition, bipolar LV pulses can increase the interaction between DNA and the membrane (Faurie et al., 2004) and improve electrotransfer efficiency (Orio et al., 2012). The combination of poring and transfer pulses could in part be responsible for the high rate of biallelic mutations observed in *OCT4* gene (85%) compared to the Namula et al. (2019) study which used only 3 poring pulses and obtained less than 5% biallelic mutations for the 15 V condition.

The concentration of CRISPR/Cas9 RNPs used for microinjection or electroporation often requires optimization to achieve optimal target disruption, where typically higher RNP concentrations being more efficient, while high concentrations can also result in increased toxicity. Cas9 protein concentrations above 100 ng/μL have usually been used for electroporation of mouse and rat zygotes in order to generate NHEJ-mediated indels or HDR-mediated nucleotide substitutions with reasonable efficiency (Chen et al., 2016; Tröder et al., 2018). Remy et al. (2017) reported 60% NHEJ and 25% knock-in efficiency in rats electroporated with 3 μM (∼480 ng/μL) Cas9 protein. One argument to use high concentrations of CRISPR/Cas9 components for genome editing is to reduce the level of mosaicism, despite the fact it may reduce embryo viability (Mehravar et al., 2019). Tanihara et al. (2019) reported that increasing Cas9 protein concentration from 20 to 100 ng/μL for cytoplasmic microinjection of porcine zygotes increased not only mutation efficiency but also the proportion of biallelic mutations. In our study, there was no difference in embryo development when 100:50 ng/μL and 200:100 ng/μL of Cas9:sgRNA were used for electroporation, providing a good range for testing and optimizing reagents for efficient gene editing.

Given that the ZP has been reported to negatively affect CRISPR/Cas9 electroporation efficiency in mouse zygotes (Qin et al., 2015; Chen et al., 2016), we tested whether large laser-drilled holes in the ZP would increase mutation rate by facilitating the flow of RNPs components into the perivitelline space of bovine zygotes. Zona drilling followed by electroporation did not affect embryo development, nor did it increase indel rates, indicating that the bovine ZP is not an obstacle for RNP components. These results are consistent with successful gene editing after RNP electroporation of zona-intact mouse and rat zygotes (Kaneko, 2017). The zona pellucida is a porous non-charged network structure and in bovine oocytes and zygotes pores range in sizes from 171 to 223 nm in diameter (Vanroose et al., 2000; Báez et al., 2019), whereas Cas9 protein has approximately a 7.5 nm hydrodynamic diameter and the sgRNA has a 5.5 nm hydrodynamic diameter (Mout et al., 2017). Thus, in bovine zygotes, the ZP does not represent a barrier to the efficient electroporation of CRISPR/Cas9 RNPs.

An important factor to consider in CRISPR/Cas9 experiments is the sgRNA efficiency. Despite not making any direct comparisons between sgRNAs in this study, we noticed differences in mutation efficiency between experiments that targeted different genes. While *ZFX* sgRNA achieved up to 37% indel mutation rate, *OCT4* sgRNA resulted in 92.3% mutations. Such differences may be due to features inherent of each individual sgRNA and/or targeted region, which may include characteristics such as GC content, purine residues position, accessibility of seed region, and secondary structure (Doench et al., 2014; Moreno-Mateos et al., 2015; Cui et al., 2018). While bioinformatic tools provide predictions of sgRNA efficiency (Cui et al., 2018; Liu et al., 2020), these predictions are not often accurate *in vivo* and thus testing multiple sgRNA is necessary for optimizing mutation efficiency, regardless of the RNP delivery method.

One-step zygote editing is often associated with high levels of mosaicism resulting from indel introduction after the first round of DNA replication (Yen et al., 2014; Sato et al., 2018; Mehravar et al., 2019). Assessment of mosaicism in preimplantation embryos is complicated given the limited amount of sample from single embryos. To circumvent this limitation, we sought to assess gene editing efficiency and embryo mosaicism using a model in which KO efficiency can be determined at the single cell level by immunostaining for the protein encoded by the targeted gene. OCT4 is expressed from the embryonic genome at morula stage, with all cells presenting positive staining at this stage in development. We previously reported that microinjection of CRISPR/Cas9 RNPs targeting *OCT4* resulted in high mutation efficiency, suppression of the OCT4 protein, as demonstrated by immunofluorescence staining, and developmental arrest at the morula stage (Daigneault et al., 2018). Interestingly, CRISPR/Cas9 RNP microinjection resulted in mosaicism of OCT4 expression in 29% of morula stage embryos. The use of the same sgRNA delivered by electroporation in this study resulted in high rate of gene editing, with most embryos (11/13) presenting biallelic mutations, and evidence of genetic mosaicism observed in only one embryo (1/13), while based on immunostaining, none of the embryos analyzed were positive for OCT4 in any of their cells (100% KO; no mosaicism). The mutation rates assessed by embryo genotyping were higher for electroporation compared to previously reported (Daigneault et al., 2018) microinjection results (92 vs. 84%, respectively). As previously reported, embryos with OCT4 mutations arrested at the morula stage, with a single embryo in this study developing to the early blastocyst stage and presenting a reduced cell number compared to controls. Overall, we show that electroporation of RNPs resulted in efficient OCT4 KO and embryo phenotypic changes consistent with lack of OCT4 function.

In conclusion, Cas9:sgRNA RNPs can be delivered efficiently by electroporation of zona-intact bovine zygotes without affecting embryo development. Electroporation of Cas9/sgRNA RNPs into bovine zygotes can result in highly efficient mutation induction, gene disruption and expected phenotypic changes. The use of electroporation for introducing gene edits in zygotes significantly simplifies the methodology for creating gene edited livestock.

## Data Availability Statement

All datasets generated for this study are included in the article/Supplementary Material, further inquiries can be directed to the corresponding author.

## Author Contributions

PR and LC conceived and designed the work. LC and JO collected and analyzed the experimental data. PR, LC, AV, and JO wrote and revised manuscript. All authors contributed to the article and approved the submitted version.

## Conflict of Interest

The authors declare that the research was conducted in the absence of any commercial or financial relationship that could be construed as a potential conflict of interest.
