# Supplementary material for: Efficient One-Step Knockout by Electroporation of Ribonucleoproteins Into Zona-Intact Bovine Embryos
Source: Front Genet. 2020 Sep 7;11:570069. doi: 10.3389/fgene.2020.570069 (PMC7504904; doi:10.3389/fgene.2020.570069)
Supplement: Supplementary file 1 [file Table_1.PDF]

Supplementary Table 1. Number of embryos evaluated in each experiment.

Experiment 1a	# oocytes analyzed - fluorescence
0V	4
10V	4
15V	8
20V	8
Experiment 1b	
0V	7
20V	6
25V	9
30V	6

Experiment 2	# presumptive zygotes	# Blastocysts
0V	114	33
15V	89	18
20V	84	14

Experiment 3	# presumptive zygotes	# Blastocysts	# embryos genotyped
Control	112	29	4
100:50 ng/uL	103	29	27
200:100 ng/uL	101	29	25

Experiment 4	# presumptive zygotes	# Blastocysts	# embryos genotyped
Control	69	23	5
Control+zona drilling	69	28	.
Intact electroporation	45	17	16
Zona drilling+electrop	98	27	25

Experiment 5 (morula development)	# presumptive zygotes	# Morulas	# embryos stained
Control	167	33	16
Control Electroporation	117	20	11
KO-OCT4	220	25	21

Experiment 5 (blast. development)	# presumptive zygotes	# Blastocysts	# embryos stained
Control	81	17	10
Control Electroporation	55	14	9
KO-OCT4	87	1	1
