# Supplementary material for: Efficient One-Step Knockout by Electroporation of Ribonucleoproteins Into Zona-Intact Bovine Embryos
Source: Front Genet. 2020 Sep 7;11:570069. doi: 10.3389/fgene.2020.570069 (PMC7504904; doi:10.3389/fgene.2020.570069)
Supplement: Supplementary file 2 [file Image_1.pdf]

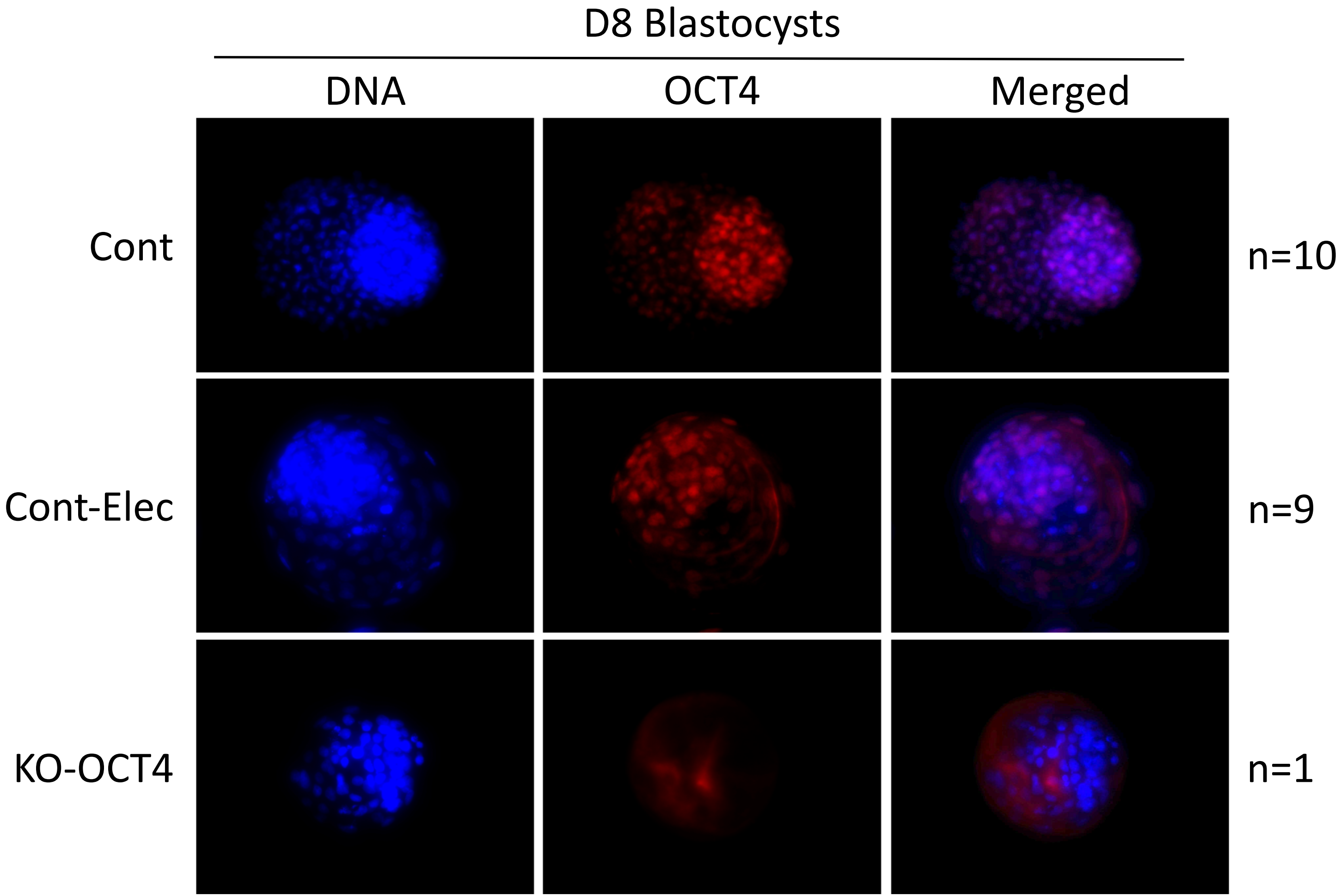

SUPLEMENTARY FIGURE 1 | Immunofluorescence analysis of OCT4 expression in blastocysts collected 192h post in vitro fertilization (D8) in control (Cont), control electroporation (Cont-Elect) and OCT4-targeting RNPs electroporation (KO-OCT4) groups.
